# Isolation of a strain of *Pseudomonas putida* capable of metabolizing anionic detergent sodium dodecyl sulfate (SDS)

**Published:** 2011-03

**Authors:** V Chaturvedi, A Kumar

**Affiliations:** School of Biotechnology, Banaras Hindu University, Varanasi-221 005, India.

**Keywords:** Biodegradation, SDS, *Pseudomonas putida* SP3, Zymography, alkyl sulfatase

## Abstract

**Background and objectives:**

Sodium Dodecyl Sulfate (SDS) is one of the most widely used anionic detergents. The present study deals with isolation and identification of SDS-degrading bacteria from a detergent contaminated pond situated in Varanasi city, India.

**Materials and Methods:**

Employing enrichment technique in minimal medium (PBM), SDS-degrading bacteria were isolated from pond water sample. Rate of degradation of SDS was studied in liquid PBM and also degradation of different concentrations of SDS was also studied to find out maximum concentration of SDS degraded by the potent isolates. Alkyl sulfatase activity (key enzyme in SDS degradation) was estimated in crude cell extracts and multiplicity of alkyl sulfatase was studied by Native PAGE Zymography. The potent isolate was identified by 16S rRNA sequence analysis.

**Results:**

Using enrichment technique in minimal medium containing SDS as a sole carbon source, initially three SDS degrading isolates were recovered. However, only one isolate, SP3, was found to be an efficient degrader of SDS. It was observed that this strain could completely metabolize 0.1% SDS in 16 h, 0.2% SDS in 20 h and 0.3% SDS in 24 h of incubation. Specific activity of alkyl sulfatase was 0.087±0.004 µmol SDS/mg protein/min and Native PAGE Zymography showed presence of alkyl sulfatase of Rf value of 0.21. This isolate was identified as *Pseudomonas putida* strain SP3.

**Conclusion:**

This is the report of isolation of SDS-degrading strain of *P. putida*, which shows high rate of SDS degradation and can degrade up to 0.3% SDS. It appears that this isolate can be exploited for bioremediation of this detergent from water systems.

## INTRODUCTION

In India, ponds are often used for washing of clothes by washer men and for bathing purposes by local people (1). In some ponds, municipal waste and wastes generated from cottage industries are also discharged. Hence, it has been observed that most of the ponds situated in Varanasi city have become eutrophic and are highly contaminated especially with high amounts of detergents (2, 3).

Sodium Dodecyl Sulfate (SDS) is an anionic detergent widely used in the manufacturing of a number of household and industrially useful products (4). Like any other chemical, SDS is discharged in water bodies like ponds and rivers (5, 6). Studies have revealed that SDS is toxic to aquatic animals such as fish, microbes like yeasts and bacteria (7–9) and also to mammals (10). So, bioremediation of this detergent was realized to be an effective method to reduce its toxicity in environments (11, 12). There have been a number of reports of isolation of SDS-degrading bacteria from different parts of the world (13–15). However, there are very few reports if any from Indian subcontinent (2, 16). It has been reported that members of the family *Pseudomonas* are capable of degrading SDS and utilizing it as a carbon source (17, 18) though bacterial strains other than *Pseudomonas* have also been reported from different parts of the world (13–15). The pathway of SDS degradation for is also well documented (10, 19). The pathway is initiated with the enzyme alkyl sulfatase which cleaves sulfate group of SDS forming 1-dodecanol (C_12_ alcohol), which is subsequently oxidized to 1-dodecanoic acid. Finally, 1-dodecanoic acid enters into - oxidation pathway and is utilized as carbon source (19).

Intensive survey revealed that there are a number of ponds in Varanasi city which are frequently used for washing and bathing purposes. As SDS is one of the widely used detergents, we became interested in screening for the presence of SDS degrading bacteria from pond water. In the present study, we have made an attempt to isolate and identify SDS degrading bacteria from a man made pond situated at Sunderpur in Varanasi city India.

## MATERIALS AND METHODS

Enrichment and isolation of SDS-degrading bacteria. For the isolation and enrichment of SDS- degrading bacteria, 1 ml of pond water was inoculated in 100 ml sterilized PBM (K_2_HPO_4_ 0.1%, KH_2_PO_4_ 0.1%, NH_4_Cl 0.1%, MgSO_4_.7H_2_O 0.02%, NaCl 0.05%, CaCl_2_ 0.002%, trace elements (FeCl_3_·6H_2_O 0.024%, CoCl_2_·6H_2_O 0.004%, CuSO_4_·5H2O 0.006%, MnCl_2_·4H_2_O 0.003%, ZnSO_4_·7H_2_O 0.031%, Na_2_MoO_4_·2H_2_O 0.003%) pH 7.5) supplemented with SDS (0.1%) in a 250 ml culture flask and incubated at 37°C and 150 rpm in a shaker (Orbitek LT, Scigenics Bioteck. Pvt. Ltd., Chennai). After 3-4 days of growth, the culture (1 ml) was transferred to fresh 100 ml PBM supplemented with SDS (0.1%). Sub- culturing was repeated for at least 3-4 times which resulted in the enrichment of putative SDS-degrading bacteria. For isolation of SDS-degrading bacteria, 0.1 ml of enrichment culture was serially diluted in PBM and was spread on PBM agar plates containing SDS (0.1%). The plates were incubated at 37°C in a BOD incubator for 2–3 days until minute colonies appeared and based on colony morphology, different SDS-degrading bacteria were isolated.

**Kinetics of SDS biodegradation**. For the study of kinetics of SDS degradation, all the test isolates were grown overnight in Luria-Bertani (LB) medium (tryptone 10%, NaCl 0.5%, yeast extracts 0.5%, pH 7.0).The cultures were centrifuged at 8,000 rpm for 5 min at room temperature, washed with PBM and then OD_600 nm_ of each isolate was adjusted to approximately 10.0 with PBM, 0.250 ml of the culture was used to inoculate 100 ml of PBM containing varying concentration of SDS (0.1 to 0.5%). The cultures were incubated at 37°C with shaking at 120 rpm. At regular intervals, samples were removed and assayed for culture OD at 600nm and residual SDS by stains all (20). All the experiments were performed in triplicate and results were expressed as Mean± standard deviation.

**Preparation of crude cell extracts**. Strain SP3 was grown in PBM containing SDS (0.1%) as a sole carbon source. Cells were harvested in mid logarithmic phase by centrifugation at 10,000 rpm for 20 min at 4°C in a Sorvall RC-5B superspeed refrigerated centrifuge (Du Pont Instruments, USA). Cell pellets were washed twice and resuspended in 10 mM Tris-HCl (pH 7.5). The cells were ruptured by sonication for a total duration of 3 min, consisting of intermittent sonication for 30 s on and 30 s off, operated at level 5 and a 25% duty cycle in a Branson Sonifier 450 (Branson Ultrasonics Corp., USA). To minimize heat inactivation of enzymes, samples were sonicated in eppendorf tubes kept on ice. Cell debris was removed by centrifugation at 12,000 rpm for 10 min at 4°C. The cell extracts were stored at -20°C prior to assay for alkyl sulfatase activity (18).

**Alkyl sulfatase activity and zymography**. Alkyl sulfatase activity in crude cell extracts was performed as per the method of Ellis et al. (18). All the experiments were performed in triplicate and results were expressed as Mean±standard deviation. To determine the multiplicity of alkyl sulfatase activities in cell extracts, polyacrylamide gel electrophoresis (PAGE) was carried out with crude cell extract (containing approx. 200 µg protein) in 6% polyacrylamide gel prepared in 0.378 M Tris-glycine buffer (pH 8.3). Electrophoresis was conducted at 100 V and at 4°C to minimize heat inactivation of enzyme. Extruded gel was incubated in developing solution containing 20 mM SDS in 0.1 M Tris-HCl (pH 7.5) at 30°C. Location of active alkyl sulfatase was revealed by the formation of white band of insoluble alcohol droplets (18). Alkyl sulfatase was characterized by Rf value, which was calculated by dividing the distance migrated by the enzyme by the distance migrated by the dye.

**Preparation of PCR products for Sequencing and Identification**. 1.5 kb fragment of 16S rDNA was amplified by universal primer (21). The PCR product was purified by Invitrogen kit (Invitrogen Corpn, CA, USA) following the instructions of manufacturer. The PCR product was eluted in Milli Q water and purity and concentration of DNA was checked by reading OD at 230, 260 and 280 nm. The purified PCR product was employed for the sequencing. The sequencing PCR reaction mix (DNA sequencing kit, Applied Biosystes, Foster city, CA, USA) contained 8.0 µL Bigdye Terminator V3.0 cycle sequencing ready reaction mixture with ApliTaq, 10ng PCR product DNA, 3.2 pmole forward primer and deionised water was added to achieve total volume of 20 µl. The thermal cycle conditions were set as; initial denaturation at 98°C for 1 min, 25 cycles of denaturation at 96°C for 10 s, annealing at 55°C for 5s and extension at 60 °C for 4 in and hold at 4°C until the purification step. Unused BigDye Terminator present in the reaction mixture was removed by Ethanol precipitation. After drying the samples, 20 µl of TSR (template suppression reagent) was added, mixed well and heated for 2 min at 95°C. Subsequently, the samples were chilled on ice and after vortexing thoroughly, centrifuged briefly in a microcentrifuge. Samples were held in ice until loading. Sequencing was performed in an ABI-PRISM 310 Genetic Analyzer (Applied Biosystems). PCR and direct sequencing were performed at least twice to determine and confirm the DNA sequences for each isolate. All the sequences were matched against nucleotide sequences present in Gen Bank using BLASTn program (22). Neighbor joining tree was prepared with the help of MEGA 4 software.

## RESULTS

**Isolation of SDS-degrading bacteria**. SDS-degrad- ing bacteria were isolated from a man made pond situated at Sundarpur in Varanasi City, India. This pond was selected because it was used extensively by local people and washer men for bathing purposes and for washing of clothes. Using enrichment tech- nique, SDS degrading bacteria were isolated from the pond water sample. A total of 3 different types on isolates were recovered from pond water. These isolates were different from each other on the basis of colony morphology, appearance, color, pigment production etc.

**Fig. 1:**
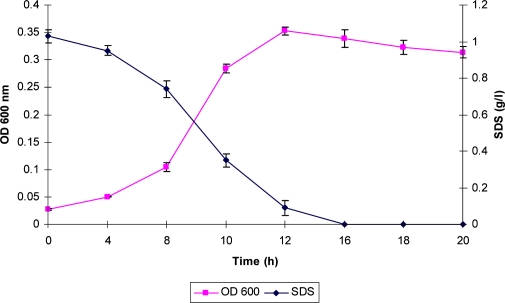
Time course study of SDS biodegradation in SP3 grown in PBM supplemented with SDS (1 g/l).

**Study of SDS degradation**. Degradation potential of SDS by these isolates was studied by their growth in PBM containing SDS as a sole carbon source. All the isolates were found to degrade and metabolize SDS as a carbon source. However, the rates of degradation of SDS were different. Only one isolates namely, SP3 was found to be efficient degrader of SDS. Other isolates showed slow and incomplete degradation of SDS. When strain SP3 was grown in PBM in presence of SDS (0.1%) complete degradation of SDS was observed in 16h of incubation with concomitant increase in the culture OD indicating utilization of this detergent as a carbon source (Fig. 1).

**Fig. 2:**
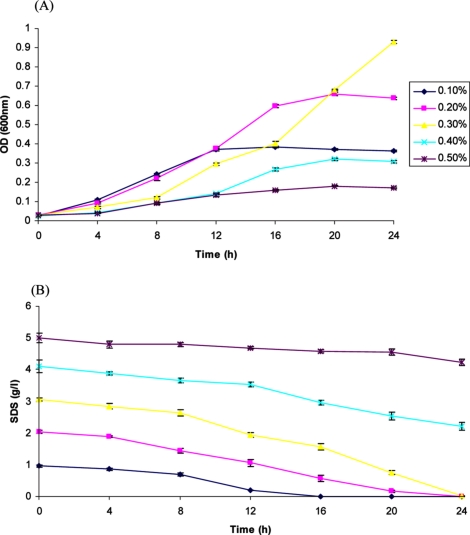
Biodegradation of higher amount of SDS in PBM by strain SP3.

**Degradation of higher amount of SDS by strain SP3**. After degradation of SDS by this isolate was confirmed, it was pertinent to study the maximum concentration of SDS that can be degraded by this strain. Biodegradation of different concentrations of SDS by strain SP3 was studied in PBM supplemented with different concentrations of SDS (w/v). It was observed that this strain could completely metabolize 0.1% SDS in 16 h, 0.2% SDS in 20 h and 0.3% SDS in 24 h with increase in culture (Fig. 2 A & B). At concentrations of 0.4% and 0.5%, the degradation was incomplete. Initially an increase in growth of the isolate as seen by an increase in culture OD with degradation of SDS but gradually absorbance declined and incomplete degradation of SDS was observed.

**Alkyl sulfatase activity and Native PAGE Zymography**. The alkyl sulfatase activity in crude cell extracts was monitored, when the isolate was in exponential phase of growth with SDS. Strain SP3 showed an alkyl sulfatase specific activity of 0.087 ± 0.004 µmol SDS/mg grown cells no alkyl sulfatase activity was observed, indicating inducible nature of the enzyme.

**Fig. 3:**
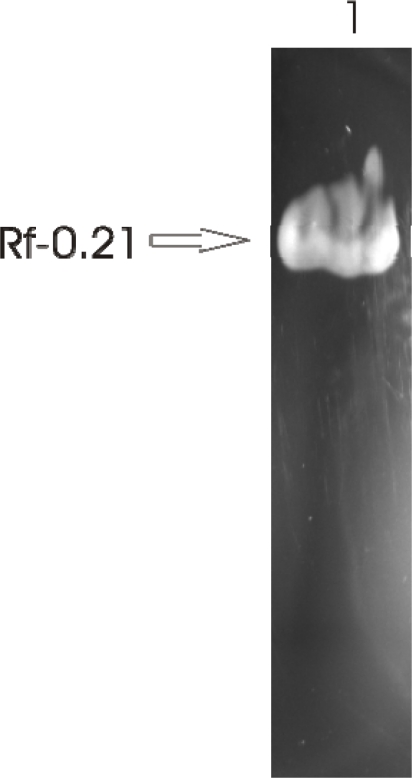
Native PAGE Zymography of alkyl sulfatase of strain SP3.

Native PAGE Zymography of alkyl sulfatase in crude cell extracts was performed. It was observed that this strain harbored an alkyl sulfatase, which had a Rf value of 0.21 (Fig. 3). Also, this alkyl sulfatase was observed throughout the growth of this strain with SDS.

**Fig. 4:**
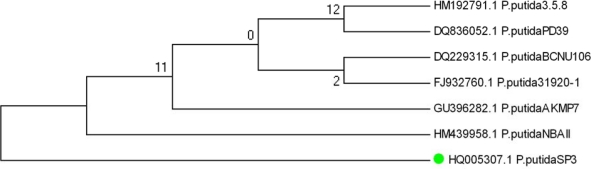
Phylogenetic tree representing *P. putida* strain SP3.

**Identification of the isolate**. This strain was identified by 16S rDNA sequencing, which is a widely used method for the identification of isolates. The 1.5 kb 16S rDNA gene was PCR amplified using universal primers and was sequenced by forward primer. The sequence was matched with NCBI database. The sequence of SP3 showed 99% similarity to *Pseudomonas putida* strain 3.5.8 (Accession No. HM192791.1), NBAII PF-4K (Accession No. HM439958.1),AKMP7 (Accession No. GU396282.1), 31920-1 (Accession No. FJ932760.1) respectively. So, this isolate was identified as *P. putida* strain SP3 and the sequence was submitted to NCBI database (Accession No. HQ005307.1) (Fig. 4).

## DISCUSSION

In the present study, we have made an attempt to isolate SDS-degrading bacteria from a detergent polluted pond situated in Sunderpur area in Varanasi city, India. This pond was used extensively for washing of clothes and also for bathing purposes. Using enrichment technique in minimal medium (PBM), three SDS-degrading bacterial isolates were recovered from the pond water. These isolates were selected on the basis certain morphological features such as color, pigment production, colony morphology etc. The rate of degradation of SDS by these isolates was studied by monitoring disappearance of SDS with time and also by measuring the growth of the isolate. It was observed that these isolates showed varying rates of SDS biodegradation. Only one isolate, namely SP3, showed appreciable level of biodegradation. This strain took 16h to completely degrade 0.1% SDS in PBM. Degradation of higher amounts of SDS was also studied. This strain was able to degrade up to 0.3% SDS in PBM. At higher concentrations of SDS, i.e. 0.4, and 0.5% SDS the degradation was incomplete. There are a number of reports of isolation of SDS-degrading bacteria from different parts of the world (12, 13, 23). However, there are very few reports of isolation of SDS-degrading bacteria from this region (2, 16). It has been observed that in most cases, SDS-degrading bacteria were recovered from activated sludge or waste water treatment plants were high concentration of detergents was observed (13–15, 24). However, in India people employ ponds for washing of clothes and bathing purposes, so high concentration of detergents has been reported in pond water (2). Use of microorganisms to degrade surfactants is promising (12). Similar to our study, Hosseini et al. (24) have isolated two strains belonging to *Pseudomonas betelli* and *Acinetobacter johnsoni* capable of metabolizing SDS. It was observed that *Acinetobacter johnsoni* was able to degrade 93.6% of 0.05% SDS within 5 days of incubation; whereas, *Pseudomonas betelli* did so to the extent of 84.6% in the same time period. The highest peak of SDS degradation occurred during the logarithmic phase of bacterial growth. Shukor et al. (15) have isolated a strain belonging to *Klebsiella oxytoca* from SDS polluted water samples from Malaysia. This isolate was able to degrade approximately 80% of 0.2% SDS after 4 days of incubation and 100% in 10 days of inoculation concomitant with increase in cellular growth. Here, our isolate appears to be more efficient than theses isolates since the rate of degradation of SDS is more in our isolate and also it can degrade higher amount of SDS.

Alkyl sulfatase activity in this isolate was also studied during exponential phase of growth with SDS as a carbon source. This isolate showed an alkyl sulfatase specific activity of 0.087 ± 0.004 µmol SDS/mg protein/min. Native PAGE zymography indicated the presence of an alkyl sulfatase of Rf value of 0.21, which was present in this isolate irrespective of growth stage. Literature survey suggests that there is much ambiguity regarding presence of alkyl sulfatase. Earlier reports suggested that bacteria capable of metabolizing SDS and related alkyl sulfate esters harbor multiple alkyl sulfatases, which are expressed during different growth stages (18, 25). On the contrary, it was shown in *Pseudomonas* ATCC19151 and *P. aeruginosa* PA01 that these isolates harbor single alkyl sulfatases SdsA (23) and SdsA1 (26) respectively, which belong to metallo-β- lacatamase family of enzymes. Moreover, it has been reported that *P. putida* strain S-313 harbors an alkyl sulfatase AtsK which belongs to α-ketoglutarate dependent dioxygenase superfamily of enzymes (27). A survey of *P. putida* KT2440 genome revealed that in addition to AtsK, this strain also harbors another alkyl sulfatase which shows low level homology to alkyl sulfatase SdsA1 belonging to *P. aeruginosa* PA01.

16S rRNA gene sequencing is one of the widely used methods of bacterial identification. This isolate was identified based on 16S rDNA sequencing. The sequence of SP3 showed 99% similarity to *Pseudomonas putida* strain 3.5.8, NBAII PF-4K, AKMP7, 31920-1 respectively. So, this isolate was identified as *P. putida* strain SP3. Until now there are several reports of isolation of SDS-degrading bacteria belonging to family *Pseudomonas* (2, 11, 12, 17, 19, 23, 24, 26, 27). However, this is the first report of isolation of a SDS-degrading strain belonging to *P. putida* from this region.

In conclusion, it appears that this strain is better adapted for rapid removal of SDS as compared to other strains isolated by different laboratories. So, this strain can be efficiently utilized for removal of detergents from detergent contaminated water systems. Additionally, a single alkyl sulfatase was observed during growth of this strain in presence of SDS. However, a search of genome sequence of *P. putida* KT2440 revealed the presence to two alkyl sulfatase genes. We feel that it would be interesting to confirm the identity of this alkyl sulfatase. We hope that this would provide new insights into degradation of SDS and related alkyl sulfate esters.
